# A Theme Issue to Celebrate Professor Robert Verpoorte’s 75th Birthday: “The Past, Current, and Future of Natural Products”

**DOI:** 10.3390/molecules26237226

**Published:** 2021-11-29

**Authors:** Yuntao Dai, Luis Francisco Salomé Abarca, Young Pyo Jang, Young Hae Choi

**Affiliations:** 1 Institute of Chinese Materia Medica, China Academy of Chinese Medical Sciences, Beijing 100700, China; ytdai@icmm.ac.cn; 2 Colegio de Postgraduados Campus Montecillo, Km 36.5 Carretera, Texcoco 56230, Mexico; luis.salome@colpos.mx; 3 College of Pharmacy, Kyung Hee University, Seoul 02447, Korea; ypjang@khu.ac.kr; 4 Natural Products Laboratory, Institute of Biology, Leiden University, Sylviusweg 72, 2333 BE Leiden, The Netherlands

Dear Colleagues,

Dr. Robert Verpoorte, Emeritus Professor at the Institute of Biology of Leiden University is among the most recognized world-wide experts in natural product chemistry. In recognition of his outstanding lifetime scientific contribution, some of his many former students and colleagues decided to honor this occasion by organizing a Special Issue of this renowned journal. 

Prof. Dr. Verpoorte was born in 1946 in Eindhoven, The Netherlands. He graduated as a pharmacist in 1967 from Leiden University, and received a master’s degree in Pharmaceutical Sciences in 1970 from the same university. He continued his studies under the supervision of Prof. Dr. A. Baerheim Svendsen (Faculty of Pharmacy, Leiden University, The Netherlands) and Prof. Dr. F. Sandberg (Faculty of Pharmacy, Stockholm University, Sweden), graduating with a PhD with his thesis on “Pharmacognostical studies of some African *Strychnos* species”. He held a position as a senior faculty member of the Faculty of Pharmaceutical Sciences, Leiden University, from 1976 until 1987, when he was appointed as Professor and Head of the Department of Pharmacognosy/Plant Cell Biotechnology at Leiden University. In 2003, his group moved to the Institute of Biology of the same university, and, until his retirement in 2011, he served as Professor and Head of the Department of Pharmacognosy/Plant Cell Biotechnology, Metabolomics Section, Institute of Biology, Leiden University. He is currently an Emeritus Professor of the Natural Products Laboratory, Institute of Biology, Leiden University.

Prof. Verpoorte was a highly prolific scientist throughout his academic career, producing over 800 scientific publications, including research papers, books, and book chapters. His research interests have been highly diverse, covering all topics related to natural products, including plant cell biotechnology, biosynthesis, metabolomics, genetic engineering, and green technology, as well as the isolation of previously unknown biologically active compounds from natural products.

Moreover, his contribution to science greatly exceeds research. He has additionally been actively involved in the organization of many courses on natural products research as a way of transmitting and sharing knowledge and experience of participants and lecturers alike. These courses were taught in many countries all over the world, and dealt with topics such as biotechnology, biosynthesis, extraction and separation, and metabolomics. Throughout these years, he has regularly delivered hundreds of lectures in conferences and scientific meetings (15–20 times per year). In addition, he has served as an editorial board member for numerous scientific journals, having been the Editor-in-Chief of the *Journal of Ethnopharmacology* (2003–2016) and currently of *Phytochemistry Reviews* (since 2001) and *Biotechnology Letters* (since 2006).

For his excellent achievements, Prof. Verpoorte has received numerous scientific distinctions, honorary doctorates, and professorships. Since his retirement, he has continued to lead an active life devoted to science, having been invited to attend many scientific meetings across the world and delivering lectures on the topics of his expertise: natural products chemistry, biotechnology, metabolomics, and green technology.

As close friends and colleagues who have been in nearly daily contact with him over the last 20 years viewing all of these remarkable scientific contributions, we felt compelled to recognize this by the publication of a Special Issue of this journal dedicated to him. 

Thus, this Special Issue has now finally been released with the help of many of his colleagues and former students as a token of our gratitude to his impressive work.

The Special Issue covers five main natural products topics: (1) chemical profiling and metabolomics, (2) separation/isolation and identification of plant specialized metabolites, (3) pharmacognosy of natural products to identify bioactive molecules from natural products, (4) novel formulation of natural products, and (5) overview of natural products as a source of bioactive molecules.

One of the new trends in natural products chemistry is the application of a holistic approach to natural products research, consisting of an untargeted analysis that can reveal whole hidden systems in organisms behind essential physiological functions (e.g., interactions between chemicals and both biotic and abiotic factors, and compound–compound interactions such as synergisms, which is the uncovering complex mechanisms of physiological roles of natural products). This approach has resulted in several scientific papers in the last decades responding to keywords, such as chemical profiling, or (the more recently coined term) metabolomics since the end of the 1990s, when this discipline was introduced in the field of life sciences.

In this Special Issue, Velasco-Azorsa et al. [1] report the application of a chemical profiling tool, an NMR-based method, to discover nematocidal metabolites in plants. Graczyk and colleagues further report an interesting approach based on the chemical profiling of the fruits of a traditional tonic plant, *Eleutherococcus senticosus,* a species that is endangered by overexploitation [2]. Their results provided valuable data for the quality control of the plant and the basis for alternatives to this plant in the future. The special issue additionally provided interesting examples of the application of diverse analytical platforms for successful chemical profiling or metabolomics studies. The potential of high-performance thin layer chromatography (HPTLC) was evaluated by Morloch and co-workers using fortified plant extracts [3]. Hyphenated techniques, such as GC-FID-MS, were additionally investigated as a tool for a metabolomics-based method to control the quality of a plum liquor by Ivanović et al., which allowed the selection and identification of 12 flavor-related volatiles as chemomarkers [4]. Another interesting NMR- and LC-MS based metabolomics approach was applied to reveal the metabolites responsible for the osmotic stress in lignan-deficient flax [5]. An interesting development was reported by Yun and colleagues, who analyzed the feasibility of using direct mass analysis; in this case direct analysis in real-time-time combined with flight-mass spectrometry (DART-TOF-MS) as a chemical screening tool for the detection of *Ephedra* alkaloids [6].

The second topic of the Special Issue is related to the basics of pharmacognosy, i.e., linking metabolites to certain bioactivities. Graziani et al. employed NMR-based chemical profiling of *Ononius diffusa* to identify cytotoxic compounds with activity against colorectal cancer cell lines. This approach was found to be much more successful, in terms of time and costs in the detection and identification of bioactive compounds, as compared to classical bioactivity-guided fractionation [7]. A similar design, integrating chemical profiling with bioactivity tests, allowed Nipun et al. [8] to identify α-glucosidase inhibitors from *Psychotria malayana* leaves using an LC-MS platform [8]. Using a more classical natural products approach, the therapeutic potential of well-known-plant-sourced compounds berberine [9], a prenyl chalcone [10], and phloridzin [11] was evaluated for their bioactivity against colorectal cancer, inflammation, and diabetes, respectively.

The isolation and identification of metabolites in plant material is undoubtedly the key step in current natural products-related research. Almost daily advances in analytical technologies are being applied to isolate natural chemicals and to elucidate their structures, reinforcing the use of natural products as a plentiful source of useful chemicals. Van der Klift and co-workers reported a technical improvement that allowed the extraction of reduced amounts of plant material (in the 1 × 10^−3^ g range) with extremely low volumes of solvents (mL range), exemplifying its successful applicability with the identification of flavones in luteola-dyed wool [12]. Lianza and colleagues provided an interesting report on the rapid identification of natural products, based on a database with an example of alkaloids from *Urceolina peruviana* [13]. The same group (Canton et al.) additionally reported the results of the application of a less common hyphenated method, centrifugal partition chromatography (CPC) with presaturation of solvent signals in ^13^C NMR, for the identification of metabolites in cosmetic samples, in which glycerin, as a residual solvent, posed a challenging analytical problem [14].

Alongside these main streams of natural products research, two multidisciplinary investigations were reported: a study on natural products formulation with ionic liquids that are known as promising green media, Kiyonga et al. [15] and a chemical ecology study related to pheromones by Pineda-Rios [16].

Finally, this Special Issue includes two interesting review papers on the current progress of the research of *Agastache* species, one of the most studied Mexican medicinal plants [17] and the chemical diversity of Datura tropane alkaloids [18].

Thus, with its 18 articles, this Special Issue covers a broad range of topics in natural products, from classical studies to new trends, with the hope of providing an overview of the past and present of research in this field, and most importantly, the promising future perspectives now possible thanks to the exciting new developments in analytical technologies.
